# Effect of changes in contractility on the index of myocardial performance in the dysfunctional left ventricle

**DOI:** 10.1186/1476-7120-4-45

**Published:** 2006-11-17

**Authors:** Steven J Lavine

**Affiliations:** 1 Department of Medicine, Division of Cardiology, Wayne State University and University of Florida/Jacksonville, Detroit, Michigan and Jacksonville, Florida, USA

## Abstract

**Background:**

The index of myocardial performance has prognostic power in patients with cardiomyopathy and following myocardial infarction. As the index of myocardial performance has been shown to be preload and afterload dependent, the effect of altering contractility on IMP and its components with left ventricular dysfunction has been incompletely delineated.

**Methods:**

Chronic left ventricular dysfunction was induced in 10 canines using coronary microsphere embolization. Each dog was instrumented and imaged with 2D echo and Doppler. At the same atrially paced rate, contractility was increased with a dobutamine infusion and then following 4 weeks of oral digoxin.

**Results:**

With chronic left ventricular dysfunction, a reduced left ventricular ejection fraction (42 ± 3%, p < 0.001) and increased index of myocardial performance (0.58 ± 0.17, p < 0.01) due to isovolumic contraction time lengthening and shortened left ventricular ejection time were noted. Dobutamine increased ejection fraction (p < 0.001), reduced left ventricular end diastolic pressure (p < 0.01), and reduced the index of myocardial performance (0.33 ± 0.17, p < 0.001) due to isovolumic contraction time, isovolumic relaxation time, and left ventricular ejection time shortening. Digoxin increased ejection fraction (p < 0.05), reduced left ventricular end diastolic pressure (p < 0.05), and reduced the index of myocardial performance (0.42 ± 0.13, p < 0.01) due to isovolumic contraction time shortening (p < 0.001). Both dobutamine and digoxin lengthened the diastolic filling period (p < 0.01).

**Conclusion:**

Increased inotropy with digoxin and dobutamine reduced the index of myocardial performance in dogs with left ventricular dysfunction. Shortened isovolumic contraction time, increased diastolic filling period, and reduced left ventricular end diastolic pressure with digoxin may provide insight into its efficacy in heart failure.

## Background

The index of myocardial performance (IMP), calculated as the sum of the isovolumic relaxation time (IRT) and isovolumic contraction time (ICT) divided by the left ventricular (LV) ejection time (LVET), has been utilized as a combined systolic-diastolic index that is prognostic in patients with dilated cardiomyopathy [1] and post myocardial infarction [2]. Its predictive value appears to be related to shorter isovolumic indices and preserved LV ejection time (LVET). In the original description of this index in patients with normal LV function and dilated cardiomyopathy, IMP was correlated with indices of systolic performance and contractility but was unrelated to heart rate, preload, and mean arterial pressure [3]. It has been further demonstrated that contractility and systolic performance were related to IMP in studies which utilize dobutamine in the normal left ventricle [4,5], in the left ventricle with reduced LV function [6,7] with contractile reserve, with the ischemic production of LV dysfunction [8], or in groups with differing LV function [1,3]. Of importance, dobutamine was demonstrated to shorten all components of IMP but specifically ICT and prolonged the diastolic filling period both in the normal ventricle [5] and in the left ventricle with dysfunction and contractile reserve [6,7].

Systematic evaluations of IMP in the normal and abnormal left ventricle have recently been performed using a canine model of chronic ischemic LV dysfunction. IMP was demonstrated to be preload and afterload dependent but not heart rate dependent [9,10]. In this model, improved systolic performance with afterload reduction with LV dysfunction was associated with shortening of the isovolumic indices and lengthening of the LVET [12]. Although clinical studies [6,7] have been performed assessing the effect of dobutamine stress on IMP with LV dysfunction, the focus has been on ischemia or contractile reserve. Limited work has been performed assessing the effect of contractility on IMP and its components with LV dysfunction without the obfuscating effects of intraventricular conduction delay or ischemic provocation [6,7].

Digoxin, an oral inotropic agent, also has beneficial symptomatic effects in heart failure patients with either reduced or preserved LV function [11]. However, the influence of digoxin on IMP is unknown and may provide some insight into its efficacy.

The goals of this study were: [1] to determine the effects of increased contractility on IMP and its components; and [2] to determine whether digoxin, a weak inotrope without an adverse survival benefit, affects IMP and its components. With LV dysfunction, we hypothesize that inotropic agents reduce the IMP by shortening isovolumic indices and possibly lengthening LVET (due to increased stroke volume) with a resultant increase in diastolic filling period. To assess this hypothesis, we used a canine model of chronic ischemic LV dysfunction producing only mild systolic dysfunction with elevated LV filling pressures where ischemia with inotropic stimulation was unlikely.

## Methods

The animals used in this study were maintained in accordance with the guidelines of the Committee on Animal Studies at Wayne State University School of Medicine and with the position of the American Heart Association on research animal use. The protocol was approved by the Animal Investigation Committee of Wayne State University. Anesthesia was induced in 10 conditioned mongrel dogs (16–24 kg) with intramuscular morphine sulfate (1.5 mg/kg) and acepromazine (1.1 mg/kg) followed in 15 minutes by 30 mg/kg of intravenous ketamine hydrochloride. Maintenance anesthesia was produced by intravenous morphine sulfate (1.5 mg/kg/hr) and pentobarbital (3 mg/kg/hr). The dogs were intubated and artificially ventilated with a Harvard respirator using room air. Using fluoroscopic guidance, two 7F high fidelity catheters (Millar Instruments) were introduced via the right carotid artery and advanced to the left ventricle and ascending aorta. A #7 multipurpose Judkins catheter was introduced through a sheath (Cordis) into the right femoral artery and advanced into the left coronary ostium. Continuous electrocardiographic monitoring was performed using lead II. At held end expiration, ECG, LV pressures, dP/dT, and central aortic pressures were obtained at 100 mm/s using an 8 channel physiologic recorder (Gould). Simultaneous 2 dimensional echocardiograms and Doppler were obtained from with the use of a phased array echocardiograph (Aloka 880). Transesophageal 4 chamber view was obtained from a 5 MHz probe placed in the mid-esophagus. Both transaortic and transmitral pulsed Doppler recordings were obtained from the LV outflow tract and from beyond the tips of the mitral leaflets in the left ventricle at 100 mm/s. Mitral regurgitation was assessed from the 4-chamber view as the size of the maximal color flow jet divided by the left atrial area.

### Induction of LV dysfunction

Ischemic LV dysfunction was induced by left main coronary artery plastic microsphere injections (58 ± 2 microns) (3M) injected in boluses of 17,500 microspheres every 5–10 minutes until the peak positive dP/dT was reduced by 25% and the LV end diastolic pressure was >12 mm Hg. LV dysfunction (LV ejection fraction = 35–40% acutely and >40% chronically) was produced in 45–60 minutes with only mild mitral regurgitation (maximal color flow jet area/left atrial area <20%). This approach ultimately leads to a model of chronic LV dysfunction where the extent of LV dysfunction may be titrated [12]. Following 45 minutes of stable hemodynamics, the above parameters were repeated. The right carotid and femoral arteries were repaired and the dogs were allowed to recover without any dog succumbing. The dogs were followed carefully for 8 weeks prior to re-study. This time period was chosen as this model has previously demonstrated interstitial and replacement fibrosis without evidence of necrosis or inflammation at 8 weeks post embolization [12].

At 8 weeks post coronary microsphere embolization, the animals were anaesthetized, intubated, instrumented, and ventilated as above. Using fluoroscopy, a #7 thermodilution catheter and a #5 bipolar pacing wire were advanced from the left internal jugular vein to the pulmonary artery and right atrium respectively. Atrial pacing commenced 10 beats above baseline rate with a PR interval <160 msec. Transthoracic and transesophageal echocardiography and Doppler were obtained as above. Continuous electrocardiographic monitoring was performed using lead II. The above hemodynamic, echocardiographic, and Doppler parameters were obtained after 10 minutes of steady state atrial pacing. Dobutamine was infused at a rate of 2–2.5 micrograms/kg/minute for 15 minutes. Care was taken to adjust the rate of infusion to ensure that atrial pacing was maintained. The above parameters were then obtained. The infusion was discontinued and the dogs were observed until hemodynamics and echocardiographic assessment of LV systolic function returned to the pre-dobutamine LV dysfunction baseline. The above parameters were then obtained as a baseline for digoxin therapy. The vessels were repaired, and the animals were recovered.

On the following day, each dog received 0.02 mg/kg/digoxin in their food daily to a maximal dose of 0.25 mg digoxin for 4 weeks [13,14]. At 1 and 2 weeks, digoxin levels were ascertained to ensure that blood levels were nontoxic. After 4 weeks of therapy, the animals were anaesthetized, intubated, ventilated, and instrumented as above. Atrial pacing commenced at the same rate as with dobutamine and hemodynamics, echocardiography and Doppler were obtained as above. The animals were then sacrificed.

### Control dogs

LV dysfunction was induced in 8 additional dogs as described above. At 8 and 12 weeks post embolization, the dogs were instrumented as described above. Hemodynamics, echocardiography, spectral, and color Doppler were obtained at baseline and with atrial pacing 10 beats above baseline. The same atrial rate was used for each dog at 8 and 12 weeks post embolization.

### Hemodynamic, echocardiographic, and transmitral Doppler measurements

For all stages and time periods, LV pressures, dP/dT, cardiac outputs, and aortic pressures were measured from the average of 3 consecutive cycles at held end expiration. The time constant of LV pressure decline was calculated using the Weiss method [15]. LV end diastolic and end systolic volumes were calculated using Simpson's Rule from the average of 3 determinations. LV ejection fraction was calculated as the difference between end diastolic volume and end systolic volume divided by end diastolic volume. LV mass was calculated by the area length method as per the recommendations of the American Society of Echo [16].

For all stages and time periods, all Doppler indices were measured from the average of 3 consecutive cycles at held end expiration. From transmitral Doppler indices, peak rapid filling velocity (E) and peak atrial filling velocity (A) were measured. The E/A ratio was calculated. The rapid filling deceleration time was calculated as the time interval from the peak rapid filling velocity to the time mitral flow decelerated to the zero baseline. The tracing was extrapolated to the zero baseline if atrial filling commenced prior to mitral flow fully decelerating to zero. Diastolic, rapid filling, and atrial filling velocity integrals were determined. Mitral regurgitation was assessed using the ratio of the maximal color flow jet area in the left atrium during systole divided by the left atrial area.

### Index of myocardial performance

From the transmitral pulsed Doppler, the time interval between the end of mitral filling and the onset of mitral filling was determined ("a"). From transaortic pulsed Doppler, the time interval from the beginning of aortic flow to the end of aortic flow was determined ("b" or LVET). The IMP can be calculated as

IMP = (a-b)/b

To determine the IRT, the time from the R wave to the end of aortic flow was subtracted from the R wave to the onset of mitral flow interval. As "a-b" represents total isovolumic time, the ICT is the difference between IRT and "a-b" [3]. The ICT, IRT, and LVET indexes were calculated to correct for differing heart rates by dividing the value by the square root of the RR interval (for non-paced comparisons).

Intraobserver variability for calculation of IMP was determined from 25 randomly selected stages. Each determination was performed 4 weeks apart. The average difference between determinations was 0.008 ± 0.002. Intraobserver and interobserver variability for LV echocardiographic volume was determined by selecting end diastolic and end systolic frames from the echocardiogram of 10 previously studied dogs. Each frame was analyzed 3 weeks apart by 2 observers (see acknowledgement). The average difference for end diastolic or end systolic volume was 2.2 ± 0.9 cc for intraobserver variability and 3.4 ± 1.7 cc for interobserver variability.

### Statistics

All data was expressed as mean ± standard deviation. Differences between a variable among stages was assessed using paired T testing. A p < 0.05 was considered to be significant.

## Results

**Figure 1:**
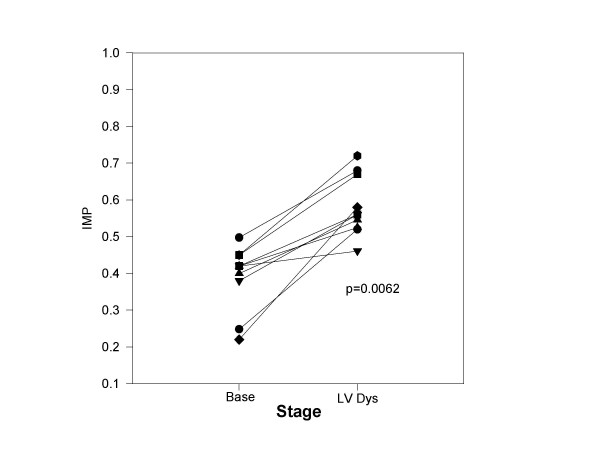
The Effect of chronic LV dysfunction on IMP: The IMP is plotted for each dog (y axis) at baseline (base) and following the development of chronic LV dysfunction (LV Dys). With chronic LV dysfunction, each dog demonstrated an increase IMP.

Table 1 summarizes the hemodynamics, LV size and function parameters, transmitral Doppler and IMP at baseline and with chronic LV dysfunction. Wi*th chronic LV dysfunction*, LV volumes, LV mass, heart rate, and LV end diastolic pressure were increased. Ejection fraction and peak +dP/dT declined. Parameters of relaxation (Tau and E/A) were also abnormal as compared to baseline. The deceleration time was also shortened suggesting reduced LV chamber compliance. This finding may be secondary to the interstitial and replacement fibrosis seen in the myocardium with this model of chronic LV dysfunction [12]. IMP was increased as compared to baseline (Figure 1) due to insignificant prolongation of IRT (p = 0.18), prolongation of ICT, and LVET shortening. At baseline, 2 of 10 dogs had trivial mitral regurgitation (jet area/left atrial area <5%). With LV dysfunction, 3 dogs had trivial or mild mitral regurgitation (jet area/left atrial area = 4%, 5%, and 8%).

**Table 1:** Hemodynamics, LV size, transmitral and transaortic Doppler, and IMP at baseline and with chronic LV dysfunction

	**Baseline**	**Chronic LVD**
**LV End Diastolic Volume (cc)**	41 ± 5	54 ± 8***
**LV End Systolic Volume (cc)**	15 ± 4	31 ± 12***
**Stroke Volume (cc)**	26 ± 3	23 ± 9
**LV Ejection fraction (%)**	63 ± 9	42 ± 3***
**LV Mass (g)**	61 ± 10	85 ± 11***
**LVSP (mm Hg)**	109 ± 14	111 ± 12
**LVEDP (mm Hg)**	6.2 ± 2.3	11.8 ± 4.9**
**Cardiac Output (l/min)**	3.1 ± 0.7	2.8 ± 0.4
**Peak +dP/dT (mm Hg/sec)**	1874 ± 244	1388 ± 206***
**Tau (msec)**	24 ± 6	39 ± 5***
**E/A**	2.1 ± 1.3	1.5 ± 0.4*
**Heart rate (beats/min)**	78 ± 12	93 ± 18*
**Deceleration Time (msec)**	217 ± 38	129 ± 36**
**ICT index (msec)**	40 ± 22	58 ± 31*
**IRT index (msec)**	46 ± 19	63 ± 29
**LV ejection time index (msec)**	226 ± 23	201 ± 24*
**IMP**	0.38 ± 0.14	0.60 ± 0.19**

**Abbreviations**: E/A = peak rapid filling velocity/peak atrial filling velocity; ICT = isovolumic contraction time; IRT = isovolumic relaxation time; IMP = Index of myocardial performance; LVSP = LV systolic pressure; LVD = LV dysfunction; LVEDP = LV end diastolic pressure.

**Figure 2:**
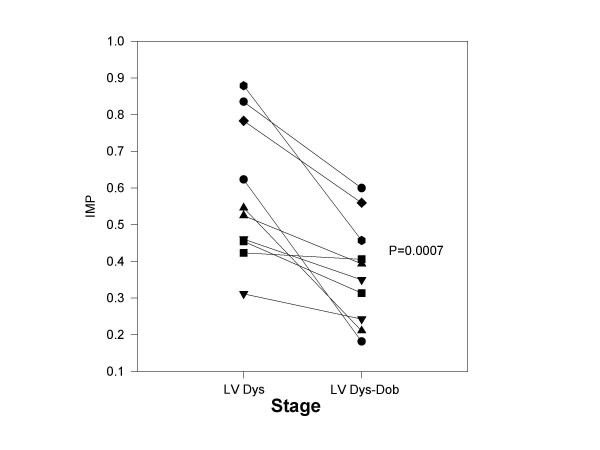
The effect of dobutamine infusion on IMP with chronic LV dysfunction: The IMP is plotted for each dog (y axis) at paced LV dysfunction (LV Dys) and following infusion of dobutamine (LV Dys-Dob). With dobutamine infusion, each dog demonstrated a decline in IMP.

**Statistics**:*p < 0.05, **p < 0.01, ***p < 0.001 vs Baseline;

Table 2 summarizes the above parameters with paced LV dysfunction and for dobutamine infusion. As expected, dobutamine increased peak +dP/dT, stroke volume, and ejection fraction with a reduction in LV end diastolic pressure. Dobutamine improved LV relaxation and LV chamber compliance (as characterized by lengthening of the deceleration time). IMP declined (Figure 2) due to marked shortening of the ICT, shortening of IRT, and shortening of LVET. Mitral regurgitation was noted in 1 of 10 dogs with dobutamine (jet area/left atrial area = 6%)

**Table 2:** Hemodynamics, parameters of LV size, transmitral and transaortic Doppler, and IMP with LV dysfunction and with dobutamine

	**Paced LV Dysfunction**	**Dobutamine**
**LV End Diastolic Volume (cc)**	55 ± 7	51 ± 8
**LV End Systolic Volume (cc)**	32 ± 7	18 ± 5***
**Stroke Volume (cc)**	23 ± 6	33 ± 7***
**LV Ejection fraction (%)**	42 ± 3	65 ± 10***
**LVSP (mm Hg)**	110 ± 12	109 ± 7
**MAP (mm Hg)**	100 ± 10	93 ± 9
**LVEDP (mm Hg)**	11.8 ± 2.4	7.5 ± 2.1**
**Peak +dP/dT (mm Hg/sec)**	1438 ± 279	2250 ± 148***
**Tau (msec)**	37 ± 6	25 ± 7***
**E (cm/s)**	81 ± 24	111 ± 16***
**A (cm/s)**	55 ± 29	54 ± 19
**E/A**	1.9 ± 0.7	2.3 ± 0.8
**Heart rate (beats/min)**	102 ± 7	102 ± 7
**DFP (msec)**	296 ± 54	364 ± 38***
**DVI (cm)**	12.6 ± 2.4	16.7 ± 2.5***
**Deceleration Time (msec)**	148 ± 30	201 ± 46**
**ICT index (msec)**	55 ± 30	17 ± 10***
**IRT index (msec)**	63 ± 25	44 ± 22*
**LV ejection time index (msec)**	204 ± 22	186 ± 28*
**IMP**	0.59 ± 0.17	0.33 ± 0.17***

**Abbreviations**: see table 1; A = peak atrial systolic velocity; DFP = diastolic filling period; DVI = diastolic velocity integral; E = peak rapid filling velocity; MAP = mean arterial pressure.

Statistics: *P < 0.05, **p < 0.01, ***p < 0.001 vs Paced LV Dysfunction

**Figure 3:**
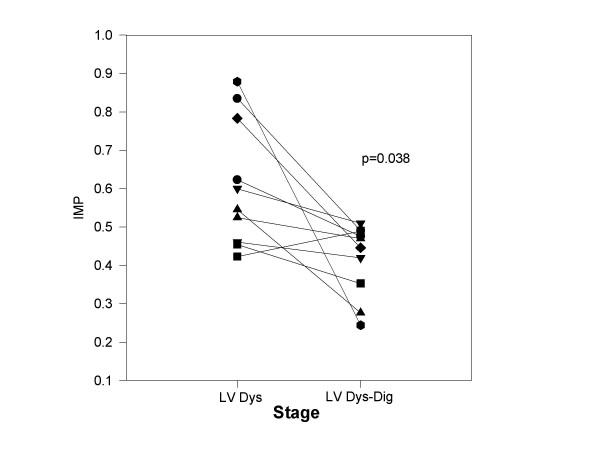
Effect of digoxin on IMP with chronic LV dysfunction. The IMP is plotted for each dog (y axis) at paced LV dysfunction (LV Dys) and following infusion of dobutamine (LV Dys-Dig). With dobutamine infusion, 13 of 14 dogs demonstrated a decline in IMP.

Table 3 summarizes the above parameters with paced LV dysfunction and with digoxin administration. Digoxin administration resulted in an increased LV ejection fraction, stroke volume, and peak +dP/dT associated with a decline in LV end diastolic pressures. The diastolic filling period prolonged without change in other diastolic function parameters measured. IMP declined in 9 of 10 dogs (Figure 3) primarily secondary to ICT shortening (>60% reduction). Mitral regurgitation was noted in 2 dogs with digoxin (jet area/left atrial area = 5%, 6%). Table 4 summarizes the hemodynamics, LV size and function parameters, transmitral Doppler and IMP 8 and 12 weeks post embolization. There were no significant differences between parameters measured at 8 and 12 weeks in control dogs strongly supporting the stability of this model. Mitral regurgitation was seen in 1 of 8 dogs at both 8 and 12 weeks (jet area/left atrial area = 5% at 8 weeks and 4% at 12 weeks post embolization).

**Table 3:** Hemodynamics, parameters of LV size, transmitral and transaortic Doppler, and IMP with LV Dysfunction and with digoxin

	**Paced LV Dysfunction**	**Digoxin**
**LV End Diastolic Volume (cc)**	54 ± 7	52 ± 8
**LV End Systolic Volume (cc)**	31 ± 10	25 ± 5*
**Stroke Volume (cc)**	23 ± 7	27 ± 7*
**LV Ejection fraction (%)**	43 ± 4	52 ± 8*
**LVSP (mm Hg)**	112 ± 14	118 ± 17
**MAP (mm Hg)**	101 ± 10	105 ± 12
**LVEDP (mm Hg)**	11.6 ± 2.1	8.6 ± 2.7*
**Peak +dP/dT (mm Hg/sec)**	1456 ± 288	1889 ± 146**
**Tau (msec)**	36 ± 7	33 ± 8
**E (cm/s)**	83 ± 22	82 ± 15
**A (cm/s)**	56 ± 17	59 ± 14
**E/A**	1.9 ± 0.8	1.7 ± 0.7
**Heart rate (beats/min)**	102 ± 7	102 ± 7
**DFP (msec)**	296 ± 54	340 ± 42**
**DVI (cm)**	12.5 ± 2.0	14.1 ± 2.4*
**Deceleration Time (msec)**	151 ± 34	149 ± 31
**ICT index (msec)**	57 ± 34	23 ± 19***
**IRT index (msec)**	61 ± 26	58 ± 19
**LV ejection time index (msec)**	204 ± 18	194 ± 19
**IMP**	0.58 ± 0.15	0.42 ± 0.13**

**Abbreviations**: see table 2

Statistics: *p < 0.05, **p < 0.001, ***p < 0.001 vs Paced LV Dysfunction

**Table 4:** Hemodynamics, parameters of LV size, transmitral and transaortic Doppler and IMP at paced LV dysfunction: 8 and 12 weeks post embolization

	**Paced LV Dysfunction-8 Weeks**	**Paced LV Dysfunction-12 Weeks**
**LV End Diastolic Volume (cc)**	54 ± 7	55 ± 6
**LV End Systolic Volume (cc)**	32 ± 12	33 ± 9
**Stroke Volume (cc)**	22 ± 8	22 ± 7
**LV Ejection fraction (%)**	43 ± 4	42 ± 4
**LVSP (mm Hg)**	116 ± 13	119 ± 21
**MAP (mm Hg)**	104 ± 15	105 ± 13
**LVEDP (mm Hg)**	11.3 ± 2.4	11.2 ± 2.0
**Peak +dP/dT (mm Hg/sec)**	1439 ± 289	1451 ± 184
**Tau (msec)**	36 ± 4	34 ± 8
**E (cm/s)**	78 ± 19	82 ± 15
**A (cm/s)**	54 ± 9	56 ± 13
**E/A**	1.8 ± 0.7	1.8 ± 0.5
**Heart rate (beats/min)**	102 ± 7	102 ± 7
**DFP (msec)**	311 ± 44	314 ± 46
**Deceleration Time (msec)**	151 ± 29	149 ± 36
**ICT index (msec)**	57 ± 25	54 ± 22
**IRT index (msec)**	58 ± 27	59 ± 19
**LV ejection time index (msec)**	201 ± 22	197 ± 25
**IMP**	0.58 ± 0.19	0.57 ± 0.17

**Abbreviations**: see table 2

## Discussion

IMP has been identified as a useful prognostic parameter following acute myocardial [2,17,18] infarction, with dilated cardiomyopathy [1], amyloid heart disease [19], and pulmonary hypertension [20]. Its prognostic capabilities appear to be mediated by increased values due to longer isovolumic indices and a shorter LVET. Despite its apparent clinical performance, IMP is preload and afterload dependent based on changes in LVET and isovolumic indices especially with LV dysfunction as previously described using a canine model of chronic ischemic LV dysfunction [9,10]. In this study, we assessed IMP and its components in this same model of chronic LV dysfunction utilizing both an intravenous and oral agent that increases contractility.

Both inotropic agents significantly increased peak +dP/dT and LV ejection fraction though the effect of digoxin was smaller. IMP was markedly reduced with dobutamine infusion with reductions in both ICT and IRT despite a reduction in LVET. The reduction in ICT was >70% and due to a combination of prolongation of time to mitral closure (12 msec) and shortening of time to onset of aortic flow (18 msec). Similarly oral digoxin for 1 month resulted in a reduction in the IMP due to a 61% reduction in ICT. Shortening of the ICT was likely related to the prolongation of the time to mitral closure (24 msec). With both agents, LV filling pressures declined and the diastolic filling period increased suggesting that reductions in total isovolumic time resulted in greater time for diastolic filling. With dobutamine, stroke volume increased and relaxation improved allowing a greater filling volume to enter a more relaxed left ventricle with lower resultant filling pressures. With digoxin, stroke volume also increased and was associated with a longer diastolic filling period resulting in a lower LV filling pressure. This finding with digoxin is intriguing as its use in patients with preserved LV function (ejection fraction >40%) has been both advocated and questioned [11,21].

### Previous literature

Using a swine model, LaCorte [8] demonstrated that IMP was related to changes in cardiac output, preload recruitable stroke work, ejection fraction, and LV stiffness with both normal LV function and following an ischemic insult. In a pediatric population, dobutamine reduced IMP and its components with the reduction in ICT being the predominant change [5]. Broberg [4] demonstrated in a mouse model of normal LV systolic function that IMP correlated with peak +dP/dT using dobutamine. Tei [3], in his original description, noted a significant relation of ICT, LVET, and IMP to peak +dP/dT, and IRT to peak -dP/dT in patients with normal LV function and cardiomyopathy patients. A systematic evaluation of contractility requires *a model of LV dysfunction *in which a positive inotrope is administered or a negative inotrope administered in a model with *normal LV function*.

However, the relationship between contractile alterations and IMP is less well delineated in the left ventricle with reduced LV function. Most information regarding the effects of contractile alterations are based on an acute ischemic episode from a normal resting LV function [8]. There are a few studies in patients with nonischemic dilated cardiomyopathy and coronary disease patients post myocardial infarction in which dobutamine reduced IMP in patients with contractile reserve but not in patients who were ischemic [6,7]. Individual IMP component analysis was performed only by Duncan [7] who noted a shortening of ICT and a lengthening of LVET in patients with nonischemic dilated cardiomyopathy and left bundle branch block undergoing dobutamine echocardiography. However, a systematic assessment of the influence of a contractile agent on IMP and its components in a model of LV dysfunction has not been evaluated. Our study fills that void and demonstrates that IMP declines with dobutamine and digoxin due to a marked reduction in ICT using a model of mild LV dysfunction. As salutary effects of digoxin have been demonstrated in several clinical trials with preserved [9] and reduced LV ejection fractions [11,22], the data in our trial may shed some light on a potential mechanism of improvement seen in the DIG trial. Not only did digoxin decrease IMP by virtue of reducing the ICT, the diastolic filling period lengthened and LV end diastolic pressure declined.

### Limitations

As this is an experimental study, the applicability of these findings and relation to intact humans is always a limitation. The general anesthesia utilized may influence the results. However, this anesthesia regimen has been utilized in multiple experimental studies [9,10,12] and has not reduced LV systolic function. Second, the use of dobutamine was always followed by digoxin administration may have a generated a series effect. However, the acute effects of dobutamine were evaluated first and the effect of digoxin was evaluated after oral therapy several weeks later. Baseline LV systolic and diastolic function parameters were virtually unchanged in control LV dysfunction dogs with a similar degree of LV dysfunction at 8 and 12 weeks post embolization. Baseline LV dysfunction may change over this time in this model but appears to be associated with significant (moderate or greater) mitral regurgitation [14]. Third, the model of LV dysfunction used to assess the IMP may not be applicable to all patients with LV dysfunction. We believe that this model is applicable as we used a coronary microsphere model of LV dysfunction which results in diffuse fibrotic changes throughout the myocardium which bears great similarity to histopathogy seen in diabetic cardiomyopathy [12,23], ischemic cardiomyopathy, and hypertensive heart disease with LV dysfunction. Finally, it is not clear that dobutamine or digoxin might have similar effects on IMP and its components with more severe LV dysfunction. As the LV ejection fraction was <40% (moderate LV dysfunction) in several animals, and the responses of IMP and ICT were uniform in most dogs, it might be reasonable to expect that inotropic stimulation with more severe LV dysfunction might produce similar responses in IMP and ICT. However, specific evaluation of dogs with varying degrees of induced LV dysfunction would be required.

## Conclusion

Though IMP has been demonstrated to be load dependent [9,10], it clearly reflects changes in the contractile state. IMP, as a single parameter indicator describing a combined systolic-diastolic index, may categorize LV function and predict prognosis post myocardial infarction or with dilated cardiomyopathy [1-3]. Its prognostic ability may very well be based on shortening of ICT and lengthening of LVET [1,2]. The major influence was on ICT in this study, and ICT has also been demonstrated to decline with afterload reduction [10]. A secondary finding was the reduction in LV end diastolic pressure associated with prolongation of diastolic filling with both digoxin and dobutamine. In particular, digoxin reduced LV end diastolic pressure to a similar degree as dobutamine but without affecting LVET and IRT. As the ejection fraction was mildly depressed secondary to coronary microsphere induced micro-infarctions and fibrosis [12], it may have direct relevance to patients with both ischemic and hypertensive etiologies of LV dysfunction. The significance of the effect of digoxin on the length of the diastolic filling period needs further investigation in patients with heart failure and preserved LV function.

In conclusion, the effect of increasing inotropic stimulation in a model of mild chronic canine LV dysfunction was determined. Increasing inotropy with digoxin and dobutamine reduced IMP primarily due to ICT shortening. Dobutamine had the additional effects of IRT and LVET shortening. Of note, a shortened ICT, increased diastolic filling period, and reduced LV end diastolic pressure with digoxin may provide insight into its efficacy in heart failure.

## List of abbreviations

ICT = isovolumic contraction time

IMP = Index of myocardial performance

IRT = isovolumic relaxation

LV = left ventricular

LVET = left ventricular ejection time

## Competing interests

The author(s) declare that they have no competing interests.

## Authors' contributions

As this is a single author paper, the author designed, acquired and analyzed the data. The author drafted and wrote the manuscript.
